# Xerostomia and Its Association with Polypharmacy and Oral Health Status Among Elderly Saudi Individuals from an Institutional Rehabilitation Center

**DOI:** 10.3390/jcm15207770

**Published:** 2026-10-09

**Authors:** Abdullah Ali H. Alzahrani

**Affiliations:** Oral and Dental Health Department, Faculty of Applied Medical Sciences, Al-Baha University, Al-Baha 65731, Saudi Arabia; aahalzahrani@bu.edu.sa; Tel.: +966-177257700 (ext. 14899)

**Keywords:** aged, dental care, oral health, polypharmacy, preventive dentistry, public health dentistry, xerostomia

## Abstract

**Background/Objectives**: The percentage of elderly Saudi individuals is expected to grow significantly in the next few decades, increasing the need for geriatric oral care. However, very little is known about multiple-drug exposure, xerostomia, and oral health among elderly Saudi individuals. Thus, this study aimed to explore the prevalence of xerostomia and its association with polypharmacy and oral health status among elderly individuals from an institutional rehabilitation center in Al-Baha Region. **Methods**: We conducted a cross-sectional study of elderly individuals who were assessed using the Summated Xerostomia Inventory (SXI) and unstimulated whole salivary sialometry (October 2024 to February 2025). Clinical oral examinations were also conducted using the World Health Organization criteria for root caries, candidiasis, mucosal lesions and ulceration, and denture stomatitis. The Oral Health Impact Profile (OHIP-14) was used to assess the oral health outcomes. **Results**: Xerostomia was found in 64.7% of the patients, and the prevalence gradually rose with the number of drugs used. Oral status was significantly poorer in xerostomic participants and was associated with higher Decayed, Missing, and Filled Teeth (DMFT) scores, more root and cervical caries, greater tooth loss and edentulism, a greater burden of oral candidiasis and mucosal and traumatic ulceration, and lower unstimulated salivary flow compared with non-xerostomic participants. Higher levels of xerostomia remained independently associated with polypharmacy (adjusted OR 3.9; 95% CI 1.4–10.6). **Conclusions**: The findings highlight the importance of routine medication review and structured preventive dental care to reduce oral side effects of salivary hypofunction. Performing a brief xerostomia screening and incorporating oral health into multimorbid elderly individuals’ medical management should be dental public health priorities.

## 1. Introduction

Saliva plays a crucial role in maintaining oral and overall health. Saliva not only protects and lubricates dentition and soft tissues, provides antimicrobial defense, buffers acid, and performs mechanical clearance, remineralization, and preservation of mucosal integrity but also facilitates mastication, deglutition, gustation, and phonation [1]. Any disruption of this protective environment will certainly have repercussions throughout the mouth. Two inter-related but conceptually distinct entities warrant differentiation: xerostomia, which is the subjective perception of oral dryness, and salivary gland hypofunction/hyposalivation, which is the objective quantification of decreased salivary output. The two are not tightly correlated, but a fraction of individuals who report experiencing oral dryness also show a reduction in salivary flow [2].

Xerostomia is diagnosed based on medical history, clinical examination of the oral tissues, and salivary tests that measure the salivary flow rate at rest and following stimulation. The salivary flow rate varies significantly based on psychological stimulation and the time of day. The average daily production of saliva in a healthy individual is between 0.5 and 1.5 L. The salivary flow rate shifts dramatically based on activity at rest (unstimulated), while sleeping, and while chewing (stimulated) (0.3 to 0.4 mL/min, virtually 0.0 mL/min, and 1.5 to 2.0 mL/min, respectively) [3]. Moreover, xerostomia may be a symptom of specific systemic conditions; it is the main symptom in certain secretory disorders such as hyposialia and asialia, which are conditions in their own right, and it is a side effect of head and neck radiotherapy and a wide range of medications [4,5,6]. Xerostomia increases the risk of not only mucositis but also acute impacts of radiation on salivary glands [7]. Evidence has revealed that the impact of chemotherapy on the oral microbiome composition could lead to xerostomia. Several chemotherapeutic drugs, including cisplatin and methotrexate, may also have toxic side effects on the salivary glands, leading to xerostomia [8].

Explorations of the dental literature have revealed that older individuals have a higher prevalence of xerostomia compared to other age groups. There is significant variation in prevalence estimates depending on the population and the measure used. However, around 30% of individuals over the age of 65 years and 40% of those over 80 years suffer from xerostomia [2]. Nonetheless, demographic aging around the world has been increasing, and morbidity related to xerostomia is accordingly on the rise, making it a priority for geriatric care [9].

Whole saliva is a biochemically complex substance containing electrolytes, bicarbonate buffer, mucins, immunoglobulins, and a variety of antimicrobial compounds such as lysozyme, lactoferrin, and histatins that neutralize plaque acid, prevent demineralization, inhibit microbial colonization, and lubricate mucosa and prostheses. The normal salivary apparatus has a significant functional reserve throughout senescence, but with concomitant systemic disease and the drugs used to treat it, elderly individuals become extremely vulnerable to salivary dysfunction. This drug- and disease-associated decrease in saliva is clinically relevant as it is often preventable or reversible, while the relatively small physiological changes in senescence are typically less dramatic [10,11].

Medication use is the most important risk factor for xerostomia in elderly individuals. Xerogenic agents are recognized drugs, such as tricyclic and modern antidepressants, antipsychotics, antihistamines, many antihypertensives, diuretics, anticholinergics, antiparkinsonian agents, opioids, and bronchodilators, which act primarily through an anticholinergic, sympathomimetic, or dehydration/electrolyte disturbance effect [12]. This population is typically exposed to multiple agents or multimorbidity at the same time. Consequently, polypharmacy, which is defined as the concomitant use of five or more drugs, is common; the more drugs that are used, the worse the perception of xerostomia and salivary flow [13]. Drug-induced xerostomia is particularly well recognized in elderly, frail, and cognitively impaired individuals living in institutional and nursing homes [13,14].

If the oral defense mechanism fails, the oral consequences are significant. Patients with affected tissues are more susceptible to dental caries, oral candidiasis, oral mucosal soreness and fissuring, halitosis, intolerance of dentures, dysgeusia, and dysphagia [15,16]. The effect of these consequences is far from trivial and is reproducible on various aspects of oral health-related quality of life (OHrQoL), including eating, comfort, and social participation [17]. Such effects can compound the pre-existing frailty of dependent elderly individuals and can contribute to their nutritional decline if they are poly-medicated.

In Saudi Arabia, the percentage of elderly individuals (>65 years old) is expected to grow significantly in the next few decades, increasing the need for geriatric oral care [9]. One study found a high level of self-reported xerostomia in elderly individuals, and chronically ill elderly individuals requiring continuous medication use have the highest risk [18]. However, there is limited evidence available relating to multiple-drug exposure and oral health beyond the main metropolitan cities for elderly Saudi individuals. Therefore, the current study aimed to explore the prevalence of xerostomia and its association with polypharmacy and oral health status among elderly individuals from an institutional rehabilitation center in Al-Baha Region.

## 2. Materials and Methods

### 2.1. Participants and Settings

This cross-sectional observational study was conducted among elderly individuals in an institutional rehabilitation center in Al-Baha Region, Kingdom of Saudi Arabia. The individuals were divided into various strata, and the stratified random sampling method was used.

### 2.2. Inclusion and Exclusion Criteria

The inclusion criteria were individuals aged 60 years or older who were ambulatory, could communicate, and were willing to be interviewed orally and have their saliva collected. The exclusion criteria were individuals with a history of head and neck radiotherapy, known primary salivary gland disorder (or Sjögren syndrome), recent acute oral infection, or the inability to collect saliva reliably. The study was thus restricted to dryness associated with current systemic medications or age-related change.

### 2.3. Medication History

Each of the participants underwent a structured interview, and their medication and medical history were obtained and compared with their current medications. Agents were stratified based on the therapeutic class (antihypertensives, antidiabetics, diuretics, antidepressants/anxiolytics, antihistamines, gastrointestinal agents, bronchodilators, and analgesics). The number of drugs taken daily was counted, and polypharmacy was defined as using five or more medicines regularly [19]. Participants were stratified into four groups based on the number of medications they were taking (1–2 medications, 3–4 medications, 5–6 medications, and ≥7 medications) to allow assessment of a dose–response gradient.

### 2.4. Assessment of Xerostomia and Salivary Flow

Subjective dryness (xerostomia) was measured using the Arabic-validated summated xerostomia inventory (SXI), which consists of five items: the frequency of dry mouth, difficulty eating dry food, dry mouth when eating a meal, difficulty swallowing certain foods, and dry lips. Each item is scored on a five-point scale (1 = never, 2 = almost never, 3 = sometimes, 4 = often, and 5 = always) [20]. The final score varies from 0 to 25, with a score of 0 to 12 classified as non-xerostomia and a score of 13 to 25 classified as xerostomia. Objective salivary function was determined by sialometry of unstimulated whole saliva, collected using the spitting method under standardized resting conditions and expressed in milliliters per minute [14]. Consistent with accepted criteria, an unstimulated whole salivary flow rate below 0.10 mL/min represented hyposalivation [3,21].

### 2.5. Clinical Oral Examination

Two calibrated examiners conducted all intra-oral assessments under standard illumination using a mouth mirror and probe. The inter-examiner variability was tested, and the weighted kappa statistic was 0.87, indicating high agreement and consistency between the results of the two assessors. Caries experience was recorded as the Decayed, Missing, and Filled Teeth (DMFT) index in accordance with World Health Organization basic-survey criteria, with the decayed, missing, and filled components enumerated separately [22]. The presence and distribution of root/cervical caries, the number of missing teeth, and complete or partial edentulism were documented alongside oral candidiasis (diagnosed clinically from its characteristic erythematous or pseudomembranous presentation), oral mucosal ulceration (traumatic, denture-related or non-specific), additional mucosal lesions (including angular cheilitis and fissured or atrophic tongue) and, among denture wearers, denture stomatitis. Moreover, the OHrQoL was measured with the 14-item Oral Health Impact Profile (OHIP-14), with higher scores denoting greater effect [23].

### 2.6. Statistical Analysis

The Statistical Package for the Social Sciences software version 20.0 (IBM, Armonk, NY, USA) was used. A value of *p* < 0.05 was considered to indicate statistical significance. Continuous variables are presented as the mean ± standard deviation, and categorical variables as frequencies and percentages. Categorical associations were tested with the Chi-square and Fisher’s exact tests, and group means were compared by the independent samples *t*-test and one-way analysis of variance. Polypharmacy and xerostomia were independently associated with each other in a multivariable binary logistic regression model adjusted for age, sex, and diabetes mellitus and expressed as odds ratios (ORs) with 95% confidence intervals (CIs). The number of medications was modeled as a continuous variable in a separate specification (adjusted for age, gender, and diabetes) to avoid collinearity with the polypharmacy category.

## 3. Results

In total, sixty-eight elderly individuals were assessed, with a mean ± SD age of −68.4 ± 6.2 years. Table 1 describes the participants’ demographic and clinical characteristics. Noticeably, hypertension and diabetes mellitus were the most common underlying conditions. Moreover, the distribution of medications that participants were taking is shown in Table 2. The findings of this study reveal that the antihypertensive group was predominant (64.7%). The percentages in Table 2 exceed 100% because most patients used more than one class of medications.

Overall, 44 out of the 68 participants (64.7%) met the inventory criterion for xerostomia. A distinct dose–response relationship emerged between daily medication count and xerostomia prevalence, ascending from 33.3% among those taking 1–2 drugs to 86.7% among those taking seven or more (*p* < 0.01). Table 3 and Figure 1 illustrate the prevalence of xerostomia by daily medication count.

The clinical findings of this study reveal that xerostomic participants frequently exhibited the hallmark signs of salivary hypofunction with a dry, glazed mucosa bearing only scant or frothy expressible saliva and minimal pooling in the floor of the mouth, together with a dorsum of the tongue that was characteristically depapillated, lobulated, and atrophic.

Salivary flow was significantly lower in xerostomic than in non-xerostomic participants, respectively (0.19 ± 0.08 and 0.33 ± 0.10 mL/min; *p* < 0.001). Moreover, objective measurements mirrored the subjective findings. The mean ± SD of unstimulated whole salivary flow declined progressively with medication burden and was lower in individuals taking 1–2 medications but higher in those taking seven or more medications, respectively (0.34 ± 0.10 mL/min and 0.17 ± 0.07 mL/min), indicating the hyposalivation threshold. Figure 2 shows the mean unstimulated salivary rate by medication count.

Xerostomic individuals in this study recorded a significantly higher mean ± SD DMFT index than those without xerostomia (*p* < 0.001). Likewise, patients with xerostomia had significantly more missing teeth and higher mean ± SD scores of the Oral Health Impact Profile (OHIP-14 Index) than those without xerostomia (*p* < 0.001). Substantially greater frequencies of root/cervical caries, oral candidiasis, and mucosal lesions were observed among xerostomic compared to non-xerostomic patients (*p* = 0.006, *p* = 0.03, and *p* = 0.029). Moreover, the xerostomic stratum commonly had mucosal ulceration and complete edentulism, but there was no statistically significant difference (*p* = 0.24 and *p* = 0.062). Among denture wearers (xerostomic, *n* = 26; non-xerostomic, *n* = 18), denture stomatitis was more than threefold more frequent in the xerostomic group (*p* = 0.046). Table 4 describes the oral health and quality-of-life outcomes and their association with xerostomia status, while Figure 3 shows a comparison of oral health outcomes with xerostomia status.

In xerostomic patients, caries characteristically involved cervical and root surfaces and the margins of existing restorations, frequently advancing to extensive coronal destruction, retained roots, and tooth loss; many such individuals were partially or wholly edentulous and reliant on removable prostheses. The attendant loss of salivary buffering, clearance, and remineralization renders the residual dentition acutely vulnerable. Moreover, soft tissue sequelae were equally conspicuous. Erythematous (atrophic) candidiasis predominated, manifesting as diffuse palatal erythema beneath dentures (denture stomatitis) and as lingual depapillation. Mucosal ulcerations, commonly traumatic or related to denture use upon a friable, poorly lubricated mucosa, and angular cheilitis were likewise more prevalent in xerostomic participants, reflecting the forfeiture of the protective and antimicrobial properties of saliva.

In the bivariate analysis, polypharmacy, age ≥ 70 years, and diabetes mellitus were each significantly associated with xerostomia, whereas gender was not (Table 5). In the multivariable logistic regression, polypharmacy remained the strongest independently associated factor after adjustment for age, gender, and diabetes (adjusted OR, 3.90; 95% CI, 1.40–10.60; *p =* 0.009); advancing age and diabetes retained positive but non-significant associations, suggesting that part of their crude effect operated through the greater medication burden that accompanies comorbidity. The model was well calibrated (Hosmer–Lemeshow χ^2^ = 4.61, df = 8, *p* = 0.80), showed acceptable discrimination (AUC = 0.79; 95% CI 0.68–0.90), and accounted for approximately one-third of the variance in xerostomia status (Nagelkerke R^2^ = 0.34; overall model χ^2^(4) = 19.8, *p* < 0.001).

## 4. Discussion

This study included 68 elderly individuals and found that xerostomia had a high prevalence (64.7%). The prevalence of xerostomia among the studied population was related to the increase in the number of medications taken by the patients; there was also a significant decline in the clinical outcomes of oral health and quality of life. All findings of the present study support the current evidence that xerostomia in elderly individuals is primarily a precursor for drug-induced side effects, not an inevitable side effect of aging [2,10,24].

The levels of xerostomia observed in the current study are not only higher than those in the 19.1% of elderly individuals in a Brazilian community, as reported by Fornari et al. [18], but also higher than some estimates of xerostomia among community-dwelling Saudi individuals [9]. This disparity is congruent with the present sample comprising older individuals, attending an institution and heavily medicated, which is precisely the patient profile associated with maximum risk. Comparable or higher rates have been reported among frail and institutionalized elderly individuals elsewhere, in whom pharmacological exposure constitutes the dominant etiological determinant [13,14,25]. The observed dose–response gradient, ranging from roughly one-third of patients receiving one or two medications to nearly nine-tenths of those receiving seven or more, recapitulates the relationship repeatedly demonstrated between medication count and both perceived dryness and measured hyposalivation [26,27]. Polypharmacy remained an independently associated factor after multivariable adjustment, in the presence of a statistically significant biological gradient and acceptable model discrimination, which strengthens the inference that the association is not merely an artifact of confounding by age or comorbidity.

A coherent mechanistic basis underlies these associations. Many of the frequently used classes of medications in this cohort, including antihypertensives, diuretics, psychotropic agents, antihistamines, and anticholinergic gastrointestinal medications, attenuate salivary secretion through anticholinergic or sympathomimetic actions or through fluid–electrolyte perturbation [28]. The cumulative “anticholinergic burden” of several concurrently administered agents plausibly accounts for the graded decline in salivary output across medication strata and for the proximity of the most heavily medicated patients to the hyposalivation threshold. The significantly reduced saliva flow rate among xerostomic participants corroborates the validity of the subjective inventory in this population, while the acknowledged imperfect concordance between symptom and sign reminds clinicians that troublesome dryness may persist despite ostensibly normal flow [29,30,31].

The clinical repercussions were substantial. Xerostomic participants displayed greater caries experience and, in particular, more root and cervical lesions, which represent the signature of salivary hypofunction, consequent upon impaired buffering, clearance, and remineralization [32]. Such caries are typically aggressive and recurrent, undermining root surfaces and restoration margins; in the present cohort, this translated into greater tooth loss and a higher prevalence of complete edentulism. The sequence exemplifies a clinically consequential cascade in medicated elderly individuals: attenuated salivary protection accelerates caries, which progresses to pulpal involvement, extraction, and, ultimately, edentulism and prosthetic dependence, whereupon persistent dryness further compromises denture retention and comfort.

The high prevalence of xerostomia observed in this study underscores the vulnerability of institutionalized, highly medicated older populations. In environments characterized by hyposalivation, the reduction in salivary protection accelerates dental caries and the degradation of dental hard tissues. Consequently, conventional preventive measures must be reinforced with aggressive, multi-targeted remineralization strategies. While traditional management appropriately relies on topical fluorides, meticulous oral hygiene, hydration, salivary substitutes, sugar-free chewing gum, and pharmacological sialagogues, contemporary dental medicine offers promising biomimetic alternatives to preserve enamel and dentin. Biomimetic hydroxyapatite has emerged as a viable agent for hard tissue preservation. Randomized clinical evidence demonstrates that home oral care using biomimetic hydroxyapatite can effectively promote the remineralization and desensitization of white spot lesions, presenting a non-invasive alternative or adjunct to conventional fluoridated toothpastes [33]. Moreover, zinc hydroxyapatite has been shown to form a protective synthetic layer over enamel and dentin, stimulating mineral deposition even when natural salivary calcium and phosphate buffering ions are deficient [34]. It must be noted, however, that while these biomimetic findings offer strong mechanistic and clinical promise for hard tissue remineralization, much of the direct evidence stems from broader clinical trials or general patient cohorts. Caution must be exercised when extrapolating these results directly to severely xerostomic or frail geriatric populations, where altered oral microenvironments and compliance issues may influence clinical efficacy.

To better contextualize these findings within older populations, the recent literature emphasizes that oral health outcomes are heavily dictated by the complexities of polypharmacy and cumulative xerogenic medication burdens [35]. Rather than evaluating medication risks purely by the number of prescribed drugs, contemporary frameworks distinguish between general polypharmacy and the specific pharmacological characteristics of individual agents. For instance, drugs possessing high anticholinergic or sympathomimetic properties present a significantly greater xerogenic risk than an equivalent number of non-xerogenic medications. A high cumulative anticholinergic burden directly impairs salivary gland function, resulting in the severe salivary dysfunction and compromised oral health outcomes frequently documented in institutionalized older adults [36]. Therefore, tailored dental preventive programs in geriatric care must integrate both advanced biomimetic remineralizing therapies and interdisciplinary medication reviews to mitigate the distinct pharmacological drivers of hyposalivation.

Oral candidiasis, mucosal lesions, and ulceration were likewise more frequent, in keeping with the antifungal, lubricating, and barrier functions of saliva, whose depletion fosters fungal overgrowth and leaves the mucosa friable and prone to traumatic and denture-related ulceration [37]. Among denture wearers, dryness undermines retention and lubrication and predisposes to denture stomatitis and soreness, consistent with prior reports linking dry mouth to prosthetic complaints and impaired function [38,39,40]. The high prevalence of diabetes in this sample, itself associated with hyposalivation, may have further amplified these effects [41,42]. Critically, participants with xerostomia also presented a greater burden of dental disease, tooth loss, mucosal conditions, and medication use, all of which may independently affect their OHrQoL. This aligns with evidence that xerostomia is no trivial complaint, exerting consistent adverse effects on eating, comfort, speech, and social interaction, and with reports linking polypharmacy-related dryness to gustatory and swallowing difficulties in elderly individuals [43]. Within a frail, multimorbid population, such burdens may further erode nutrition and well-being.

The findings of this study carry tangible clinical implications. Because medication represents the foremost modifiable driver of dry mouth in later life, collaborative medication review identifying xerogenic agents, rationalizing or substituting them where clinically justifiable, and forestalling prescribing cascades warrants priority [44]. Dental management should center on intensified prevention: topical fluoride and scrupulous oral hygiene to counter root caries, antifungal and denture hygiene measures where indicated, and symptomatic relief through frequent hydration, salivary substitutes, sugar-free chewing gum to stimulate residual secretion and, in selected patients, pharmacological sialagogues [45]. Structured dental recall holds particular value for poly-medicated elderly individuals.

The present study has some limitations. First, the cross-sectional design with a sample of 68 patients may limit the generalizability of the findings and statistical power. Second, the subjective inventory used to define xerostomia, as well as unmeasured potential confounding factors such as smoking, hydration status, and dosage and duration of treatment, may have affected the associations observed. Third, while diabetes mellitus was included as a covariate in our multivariable analysis, our study utilized a binary classification for this condition. We lacked detailed clinical data regarding diabetes type, disease duration, longitudinal HbA1c levels, and specific pharmacological management. Consequently, we could not fully account for variations in metabolic control, which represents a limitation of our confounding adjustment. Fourth, the absence of a formal assessment of xerogenic or anticholinergic burden may also be a limitation. Lastly, recruitment of clinic attendees may have inflated prevalence. On the other hand, this study has some strengths: all subjects were recruited from one institutional setting, thus ensuring a homogeneous sample, and this study offers insights into the association between the prevalence of xerostomia and polypharmacy among the studied population.

## 5. Conclusions

This study reveals an association between polypharmacy, xerostomia, reduced salivary flow, and several adverse oral health outcomes among elderly individuals in an institutional rehabilitation center in Al-Baha Region. The findings have direct translational implications, as the medication burden may potentially be modified in selected patients, but medication reduction or substitution depends on the underlying systemic conditions and should occur within appropriate multidisciplinary medical management. Thus, we suggest routine medication review by a physician and dentist, increasing clinical awareness for elderly individuals taking multiple medications and consequently developing dry mouth, and prevention-oriented structured dental care to reduce the oral side effects of salivary hypofunction. We make the following suggestions for future research: (1) Larger, multi-center and longitudinal investigations, ideally incorporating stimulated sialometry and formal anticholinergic burden scoring, are required to corroborate and extend these regional findings. (2) Brief xerostomia screening and incorporation of salivary assessment into geriatric dental care, as well as incorporation of oral health into medical management of multimorbid elderly individuals, should be dental public health priorities as this group continues to grow in Saudi Arabia. (3) A systematic review with meta-analysis comparing these regional findings with those of other countries would help establish stronger evidence.

## Figures and Tables

**Figure 1:**
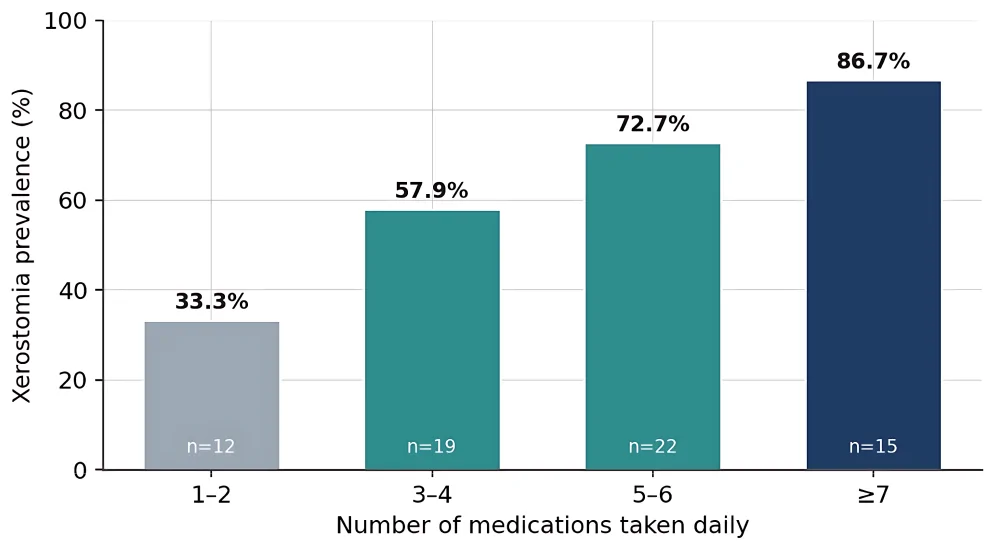
Prevalence of xerostomia rose progressively with the number of medications taken daily.

**Figure 2:**
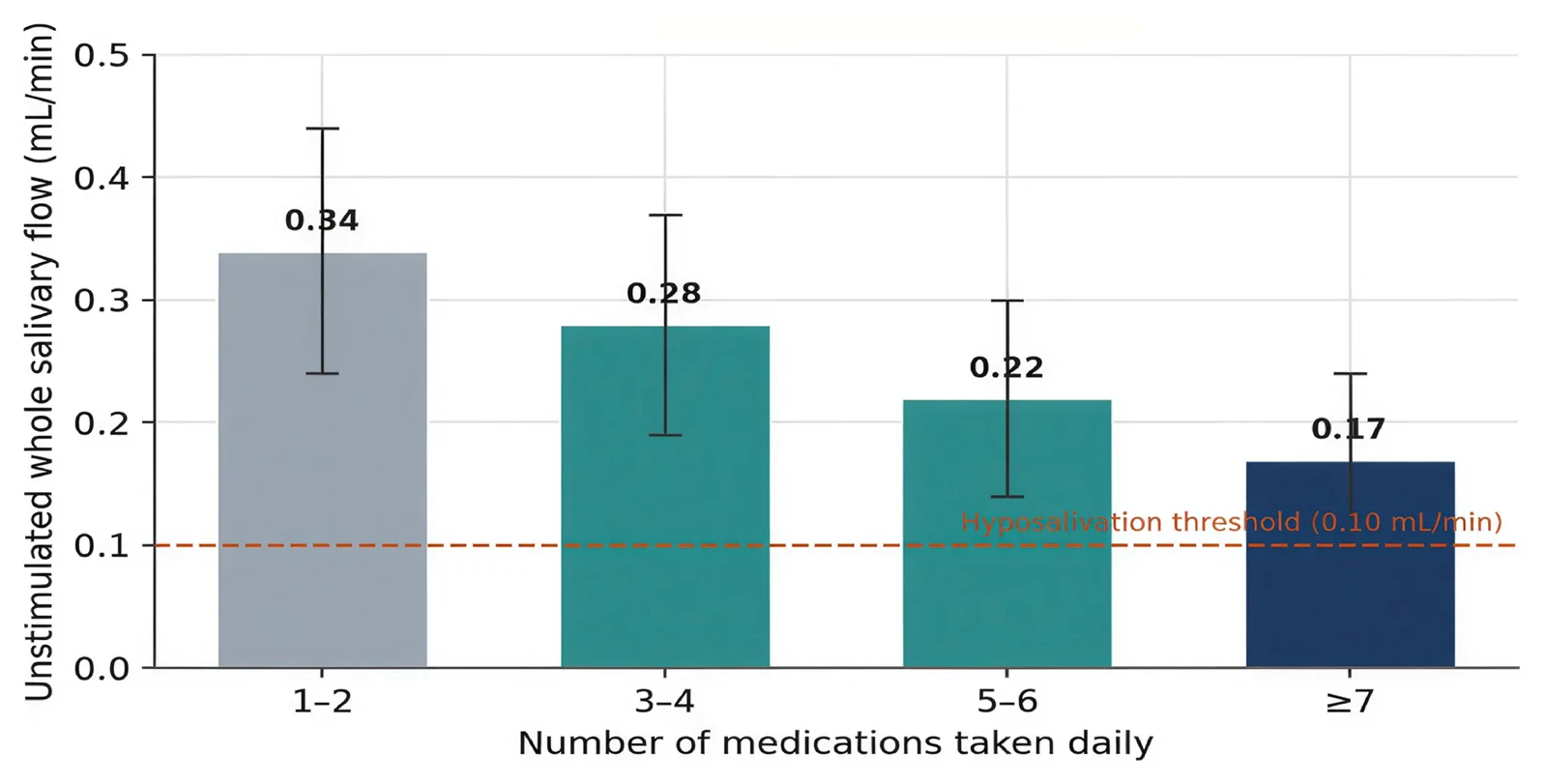
Mean unstimulated salivary flow rate decreased with increasing medication burden; error bars represent standard deviation. Dashed line marks 0.10 mL/min hyposalivation threshold.

**Figure 3:**
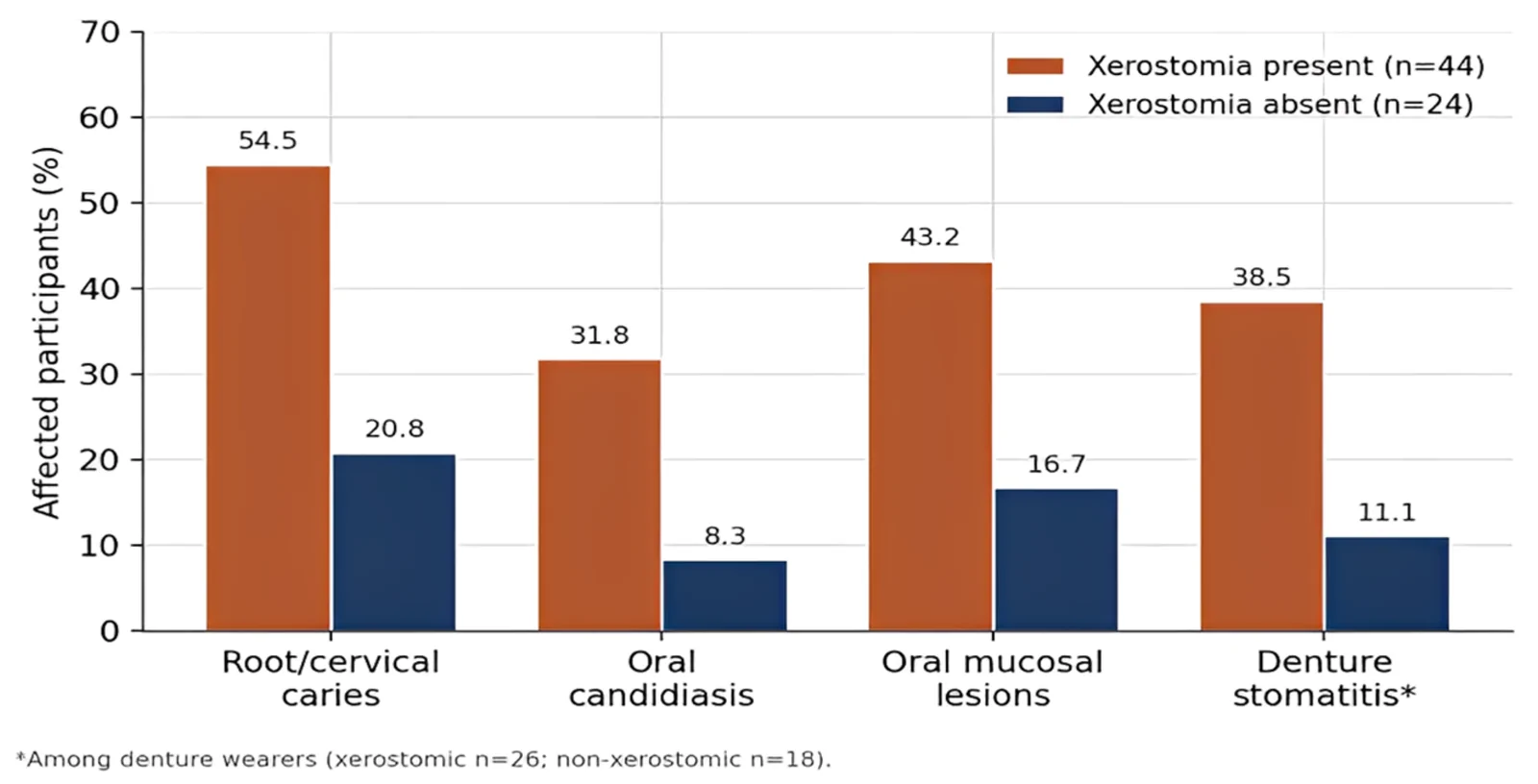
The frequency of adverse oral health outcomes was consistently higher among participants with xerostomia than among those without.

**Table 1:** Participants’ demographic and clinical characteristics.

Characteristic	*n* (%)
Gender: male and female, respectively	37 (54.4) and 31 (45.6)
Polypharmacy (≥5 medications)	37 (54.4)
Hypertension	44 (64.7)
Denture wearers	26 (38.2)
Diabetes mellitus	44 (64.7)
Hyposalivation (unstimulated whole saliva flow rate < 0.10 mL/min)	18 (26.5)

**Table 2:** Distribution of medications that participants were taking.

Medication	*n* (%)
Antihypertensives	44 (64.7)
Antidiabetic agents (oral/insulin)	29 (42.6)
Diuretics	21 (30.9)
Gastrointestinal agents (proton pump inhibitor (PPI)/anticholinergic)	18 (26.5)
Antidepressants/anxiolytics	16 (23.5)
Antihistamines	13 (19.1)
Analgesics/opioids	10 (14.7)
Bronchodilators	8 (11.8)

**Table 3:** Prevalence of xerostomia by daily medication count.

Medication Group	*n* (%)	Xerostomia Present *n* (%)	Xerostomia Absent *n* (%)	*p*-Value
1–2 medications	12 (17.7)	4 (33.3)	8 (66.7)	<0.01
3–4 medications	19 (27.9)	11 (57.9)	8 (42.1)	
5–6 medications	22 (32.4)	16 (72.7)	6 (27.3)	
≥7 medications	15 (22)	13 (86.7)	2 (13.3)	
Total	68 (100)	44 (64.7)	24 (35.3)	

**Table 4:** Oral health-related quality of life outcomes and their association with xerostomia status.

Variable	Xerostomia Present (*n* = 44)	Xerostomia Absent (*n* = 24)	*p*-Value
DMFT (mean ± SD)	19.2 ± 4.6	13.8 ± 4.1	<0.001 ^1^
Missing teeth (mean ± SD)	11.4 ± 5.2	7.9 ± 4.8	0.008 ^1^
OHIP-14 score (mean ± SD)	18.7 ± 6.3	9.4 ± 5.1	<0.001 ^1^
Root/cervical caries, *n* (%)	24 (54.5)	5 (20.8)	0.006 ^1^
Complete edentulism, *n* (%)	13 (29.5)	4 (16.7)	0.24
Oral candidiasis, *n* (%)	14 (31.8)	2 (8.3)	0.030 ^1^
Oral mucosal ulceration, *n* (%)	12 (27.3)	2 (8.3)	0.062
Oral mucosal lesions, *n* (%)	19 (43.2)	4 (16.7)	0.029 ^1^
Denture stomatitis, *n* (%)	10 (38.5)	2 (11.1)	0.046 ^1^

^1^ *p*-value is statistically significant.

**Table 5:** Binary logistic regression: crude and adjusted odds ratios of factors related to xerostomia.

Variable	Crude OR (95% CI)	*p*-Value	Adjusted OR (95% CI)	*p*-Value
Polypharmacy, ≥5 vs. <5 medications	3.87 (1.36–11.01)	0.011 ^1^	3.90 (1.40–10.60)	0.009 ^1^
Age, ≥70 vs. <70 years	3.60 (1.24–10.44)	0.016 ^1^	2.86 (0.93–8.80)	0.068
Diabetes mellitus, present vs. absent	3.29 (1.11–9.71)	0.031 ^1^	2.58 (0.82–8.12)	0.104
Gender, female vs. male	0.76 (0.28–2.05)	0.59	0.68 (0.23–2.02)	0.49
Number of medications (per drug) *	1.41 (1.14–1.74)	0.002 ^1^	1.38 (1.10–1.73)	0.005 ^1^

^1^ *p*-value is statistically significant. OR, odds ratio; CI, confidence interval. * Number of medications modeled as a continuous variable in a separate specification (adjusted for age, gender, and diabetes) to avoid collinearity with the polypharmacy category.

## Data Availability

The raw data supporting the conclusions of this article will be made available by the author upon request.

## Abbreviations

The following abbreviations are used in this manuscript:

OHIP-14	Oral Health Impact Profile Index
SXI	Summated Xerostomia Inventory
OR	Odds Ratio
CI	Confidence Interval
OHrQoL	Oral Health-Related Quality of Life
DMFT	Decayed, Missing, and Filled Teeth
Mean ± SD	Mean ± Standard Deviation
